# Draft genome sequence of a carbapenem-resistant *Pseudomonas aeruginosa* strain PA1, a clinical isolate from Bangladesh

**DOI:** 10.1128/mra.01211-25

**Published:** 2026-02-18

**Authors:** Fahd B. Zahed, Tangerul A. Jepu, M. Ismail Hossain, Fatema T. Zohora, Tasmim A. Saleh, Nusrat U. A. Saleh, M. Salah Uddin, Muktadir S. Hossain

**Affiliations:** 1 Department of Biochemistry and Microbiology, North South University 54495, Dhaka, Bangladesh; 2 Core Research Facility, School of Health and Life Sciences, North South University 54495, Dhaka, Bangladesh; Fluxus Inc., Sunnyvale, California, USA

**Keywords:** antibiotic resistance, virulence factors

## Abstract

*Pseudomonas aeruginosa* strain PA1, a multidrug-resistant opportunistic pathogen that was isolated from Dhaka, Bangladesh, has a 6.4 Mbp draft genome. The key features include the presence of multiple virulence genes and a number of antibiotic-resistance genes. PA1 belongs to ST1650, with >99.1% ANI to PAO1.

## ANNOUNCEMENT

*Pseudomonas aeruginosa*, a World Health Organization critical-priority pathogen (1), poses significant challenges in clinical settings due to its intrinsic multidrug resistance and biofilm-mediated persistence. In this study, *P. aeruginosa* PA1 was isolated from a pediatric patient by Dr. Samir Saha of the Child Health Research Foundation, Dhaka, Bangladesh.

PA1 was cultured on cetrimide agar plates at 37°C for 12 h. For liquid culture, a single colony from a cetrimide agar plate was picked and mixed with Luria-Bertani (LB) medium, followed by incubation at 37°C with 150 rpm for 16 h. For genomic DNA isolation, 5 mL of LB culture was used. The Illumina NovaSeq 6000 platform was used to sequence the genomic DNA of *Pseudomonas aeruginosa* PA1 after it was isolated using the Wizard Genomic DNA Purification Kit (Promega). The purified DNA concentration was 528.9 ng/µL, and the A_260_/A_280_ ratio was 1.8 as determined by Nanodrop. The Nextera XT DNA Library Preparation Kit (Illumina) was used to create the libraries. A quality evaluation using FastQC (Galaxy Version 0.74+galaxy0) is conducted on the paired-end sequencing data (151 bp read length), comprising 13,020,721 total sequences and 1.9 Gbp, in order to examine metrics like GC content, read length distribution, and adapter presence. Before *de novo* assembly with SPAdes (Galaxy Version 3.15.4+galaxy1), adapters and low-quality bases were cut using Trimmomatic (Galaxy Version 0.39+galaxy2) (2) generating a 6.4 Mbp draft genome (40 contigs, N50:510570) with 66.42% GC content (Table 1). The final genome coverage was 305.1×. The genome was annotated using the NCBI Prokaryotic Genome Annotation Pipeline (PGAP) software revision 6.10, which predicted 5,894 coding sequences, 58 tRNAs, and 3 rRNAs. Virulence determinants by using Virulence Factor Database (3) included adherence-associated flagellar genes and type IV pili genes, antiphagocytic alginate biosynthesis regulator genes, and genes involved in the iron acquisition systems. Quorum sensing genes, genes for tissue-degrading enzymes, and toxin genes were also identified, alongside type III and type VI secretion systems. Antimicrobial resistance (AMR) analysis across four databases (Resfinder, AMRfinder, CARD, NCBI) (4) confirmed chromosomal *pdc-3* (carbapenem resistance), *blaOXA* (β-lactam resistance), and *catB* (chloramphenicol resistance). The AMR profile was determined using the Kirby-Bauer disk diffusion method, and results were interpreted according to the Clinical and Laboratory Standards Institute (CLSI) guidelines, with resistant phenotypes defined by the following diameter of the zone of inhibition: meropenem (0.0 mm) and imipenem (0.0 mm), confirming that the PA1 strain is carbapenem-resistant. PHASTER detected four intact prophages encoding integrases and phage tail proteins (5), while CRISPRCasFinder (6) identified a Type III-U CRISPR-Cas system. Multilocus sequence typing was carried out using PubMLST (7), and PA1 is classified as ST1650, a lineage associated with nosocomial outbreaks in South Asia. For each software, the default parameters were used except as otherwise noted.

**TABLE 1:** Genome information of *Pseudomonas aeruginosa* (PA1) (8)

Features	*Pseudomonas aeruginosa,* PA1	*Pseudomonas aeruginosa,* PAO1 (Ref)
Genome size (bp)	6.4 Mbp (6,444,127 bp)	6.2 Mbp (6,264,404 bp)
DNA G+C (%)	66.42	66.56
Total contig	40	1
Total genes	6,016	5,757
rRNAs	3	12
tRNAs	58	73
tmRNA	1	1

## Data Availability

This whole genome shotgun project of *Pseudomonas aeruginosa* PA1 has been deposited at GenBank under the accession number JBQPXR000000000. The associated BioProject and BioSample accession numbers are PRJNA1299115 and SAMN50294652, respectively. The accession number of the publicly available raw data of the PA1 genome sequence is SRR35257096. The GenBank accession number of the reference genome *Pseudomonas aeruginosa*, PAO1 is AE004091.2.
